# Longitudinal SARS-CoV-2 Testing among the Unvaccinated Is Punctuated by Intermittent Positivity and Variable Rates of Increasing Cycle Threshold Values

**DOI:** 10.1128/spectrum.02715-21

**Published:** 2022-03-22

**Authors:** Shawn E. Hawken, Subhashini A. Sellers, Jason R. Smedberg, Jeremy D. Ward, Avian M. Elliott, Herbert C. Whinna, William A. Fischer, Melissa B. Miller

**Affiliations:** a Clinical Microbiology Laboratory, McLendon Clinical Laboratories, UNC Medical Center, Chapel Hill, North Carolina, USA; b Department of Medicine, Division of Pulmonary Diseases and Critical Care Medicine, School of Medicine, University of North Carolina at Chapel Hill, Chapel Hill, North Carolina, USA; c Department of Pathology and Laboratory Medicine, School of Medicine, University of North Carolina at Chapel Hill, Chapel Hill, North Carolina, USA; Labcorp

**Keywords:** SARS-CoV-2, cycle threshold, infectivity, longitudinal positive

## Abstract

The coronavirus disease 2019 (COVID-19) pandemic is complicated by cases of vaccine breakthrough and reinfection and widespread transmission of variants of concern (VOCs). Consequently, the need to interpret longitudinal positive severe acute respiratory syndrome coronavirus 2 (SARS-CoV-2) tests is crucial in guiding clinical decisions regarding infection control precautions and treatment. Although diagnostic real-time reverse transcription (RT)-PCR tests yield *C_T_* values that are inversely correlated with RNA quantity, these tests are only approved for qualitative interpretation. In this study, we performed a retrospective review of 72,217 SARS-CoV-2 positive tests and identified 264 patients with longitudinal positivity prior to vaccination and VOC circulation. Patients with longitudinal positivity fell into two categories: short-term (207, 78%) or prolonged (57, 22%) positivity, defined as ≤28 (range, 1 to 28; median, 16) days and >28 (range, 29 to 152; median, 41) days, respectively. In general, *C_T_* values increased over time in both groups; however, 11 short-term-positive patients had greater amounts of RNA detected at their terminal test than at the first positive test, and 6 patients had RNA detected at *C_T_* values of <35 at least 40 days after initial infection. Oscillating positive and negative results occurred in both groups, although oscillation was seen three times more frequently in prolonged-positive patients. Patients with prolonged positivity had diverse clinical characteristics but were often critically ill and were discharged to high-level care or deceased (22%). Overall, this study demonstrates that caution must be emphasized when interpreting *C_T_* values as a proxy for infectivity, a predictor of severity, or a guide for patient care decisions in the absence of additional clinical context, particularly among the unvaccinated population.

**IMPORTANCE** We describe the duration of positivity and the COVID-19 treatment and outcome characteristics of an unvaccinated population of patients with prolonged SARS-CoV-2 positivity. This investigation serves to highlight challenges in using *C_T_* values to guide clinical decisions among unvaccinated individuals.

## INTRODUCTION

The global burden of morbidity and mortality caused by severe acute respiratory syndrome coronavirus 2 (SARS-CoV-2) is concentrated among vulnerable patients with comorbidities (1). While most patients test negative after 14 days, cases where patients remain RNA positive months later have been observed (2–5). The recent emergence of vaccine breakthrough cases, reinfections, and widespread transmission of variants of concern (VOCs), such as World Health Organization lineages Delta and Omicron, which appear to rise to higher viral titers, underscore the clinical imperative to interpret positive SARS-CoV-2 results from the same patient over time. Unfortunately, current real-time reverse transcription (RT)-PCR clinical diagnostic tests are not quantitative (6, 7). While real-time RT-PCRs yield cycle threshold (*C_T_*) values that are inversely correlated with the amount of RNA present in a sample, they are influenced by patient, specimen, and diagnostic test characteristics (8, 9).

The debate over whether and how to use *C_T_* values clinically is underscored by recent statements from professional societies, including the joint statement from the Infectious Diseases Society of America (IDSA) and the Association for Molecular Pathology (AMP) and a statement from the College of American Pathologist (CAP), in response to growing interest in using *C_T_* values as a surrogate measure of a patient’s viral load (8, 9). Proponents of using *C_T_* values to guide clinical decisions infer *C_T_* values as a snapshot of the viral burden in a patient’s sample, and there is interest in clinical evaluation of *C_T_* trends to discern the clinical and epidemiological relevance of a positive test (9, 10). Unfortunately, the observation that a subset of patients will test positive, typically with a higher *C_T_* value, several months after initial infection is a challenge clinically, since it is often unclear whether these patients are still infectious and what distinguishes these patients from the majority of SARS-CoV-2 patients who instead convert to a negative test weeks after infection (3–5). Here, we sought to describe the natural history of SARS-CoV-2 test results, including *C_T_* values, among patients who tested positive multiple times at our institution in a time period prior to vaccine rollout and VOC circulation. Although viral and human population dynamics have changed with the emergence of highly contagious VOCs Delta and Omicron, as well as increased vaccine uptake, at the time of this writing, 25% of the eligible U.S. population and 41% of the global population remain unvaccinated. The unvaccinated population is two times more likely to require hospitalization and seven times more likely to die of coronavirus disease 2019 (COVID-19) than vaccinated persons (11, 12). We describe the duration of positivity and the COVID-19 treatment and outcome characteristics of an unvaccinated population of patients with prolonged SARS-CoV-2 positivity. This investigation serves to highlight challenges in using *C_T_* values to guide clinical decisions among unvaccinated individuals.

## RESULTS

### Summary of SARS-CoV-2 testing during study.

**FIG 1:**
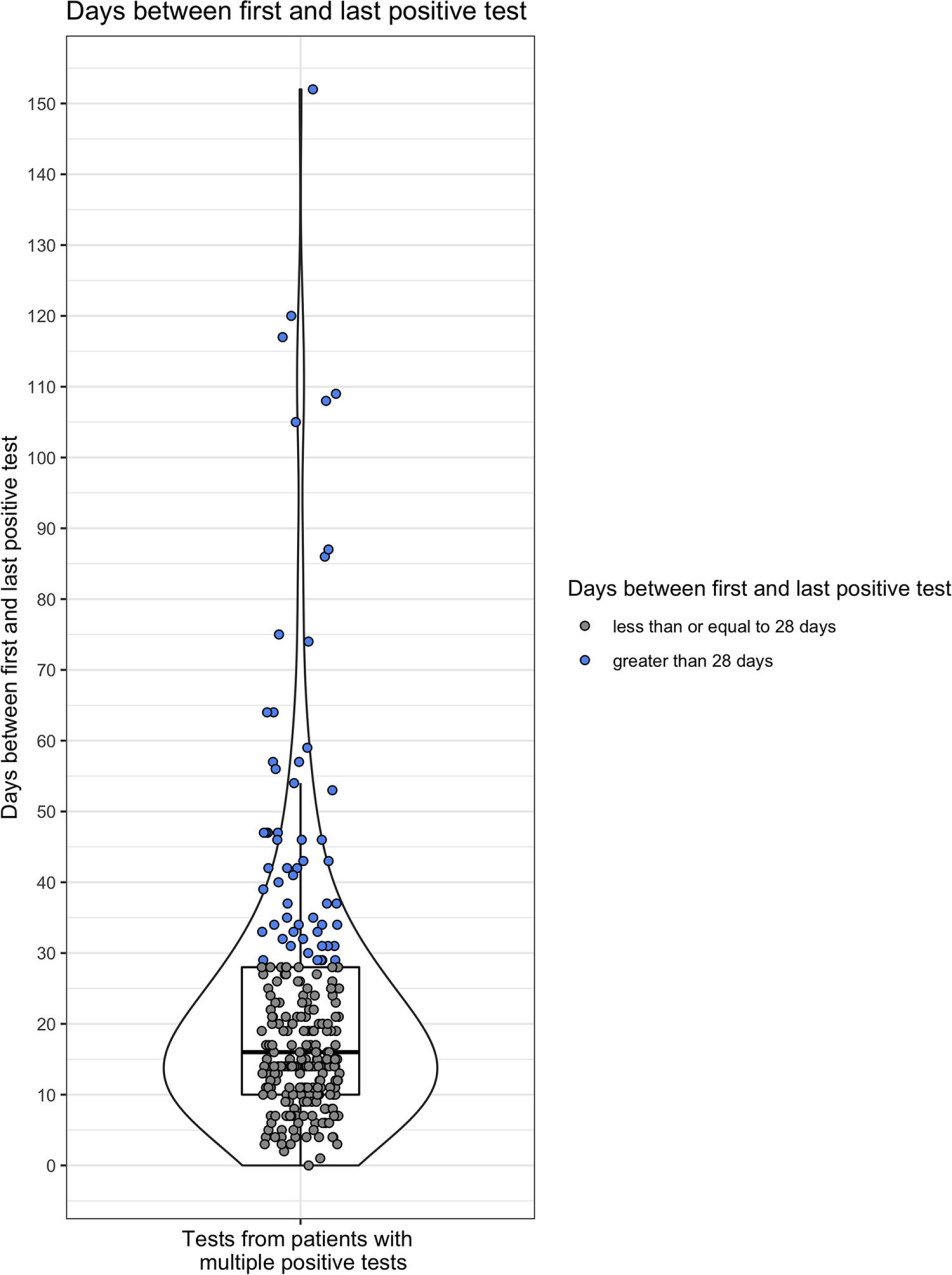
Summary of time between first and last positive tests for patients with multiple positive tests. *y* axis indicates days between first and last positive tests, and individual dots indicate individual patients. Blue indicates prolonged-positive patient defined at natural breakpoint of >3rd quartile duration, and gray indicates short-term-positive patient. Violin-and-box plot indicates overall distribution of days between first and last positive tests.

Between 16 March and 15 October 2020, our hospital performed 72,217 real-time RT-PCR tests for SARS-CoV-2, including 4,609 positive and 67,608 negative tests, across six different specimen types, including 67,612 (93%) nasopharyngeal swab samples. Real-time RT-PCR tests were performed across four platforms: 18,057 on an emergency use authorization (EUA) laboratory-developed test (LDT), 7,014 on the Cepheid Xpert, 23,983 on the Abbott Alinity m, and 23,163 on the Abbott RealTime m2000. Among the 58,348 patients tested, 9,682 (16.6%) were tested multiple times and 264 (0.45%) patients had more than one positive test (Fig. S1 in the supplemental material). Among 264 patients with multiple positive tests, the median interval between the first and last positive test was 16 days (range, 1 to 152), with 118 (44.7%) patients reverting to negative within 14 days of an initial positive result. We took advantage of a natural breakpoint in the distribution of durations of positivity to define prolonged positivity as more than the 3rd quartile (28 days) of duration between the first and last positive tests (Fig. 1). Prolonged SARS-CoV-2 positivity was observed in 57 patients, who remained positive for 29 to 152 (median, 41) days.

### Equivalency of *C_T_* values across a range of SARS-CoV-2 RNA levels and testing platforms demonstrates that normalization between platforms is not required.

Differences in real-time RT-PCR assay parameters across the testing platforms could result in different *C_T_* values being reported, thus prohibiting the direct comparison of *C_T_* value results from different testing platforms. To assess the extent that this confounds a comparison of *C_T_* values and determine whether normalization is required, we performed real-time RT-PCR on pools of positive specimens across a range of *C_T_* values, including those close to the limit of detection, where high variation could be more pronounced or account for the difference between positive and negative test results (Fig. S3). Linear regression analysis showed platform to be a poor predictor of *C_T_* value, accounting for less than 2% of the variation in *C_T_* values (*F*_2,78_ = 0.25, *P* = 0.78). Triplicate pools tested across platforms showed limited *C_T_* variation, with a maximum *C_T_* difference across platforms of 2.4 cycles and maximum standard deviation of 1.6 cycles separately across the three test days (Fig. S3). Combining triplicate real-time RT-PCRs and test days, the maximum interplatform *C_T_* differences were small for all pools (for ranges of *C_T_* values of 15 to 20, 25 to 30, and 33 to 35, the differences were 1.5 cycles, 2.18 cycles, and 0.69 cycles, respectively) (Table S1). Overall, these results show high reproducibility and limited variation across instruments, supporting similarity of *C_T_* values across the instruments used in this study and indicating that comparison of *C_T_* values between these assays is valid.

### Diagnostic testing characteristics of patients with multiple SARS-CoV-2 positive tests.

Among the 207 patients with multiple positive tests who did not have prolonged positivity, 17 (8.2%) had patterns of intermittent positivity with at least one negative test (range, 0 to 3 negative tests) between their first and last positive test. This pattern was observed over 3 times more frequently among the 57 prolonged-positive patients, 16 of whom (28%) had intermittent positivity (*P* < 0.001).

**FIG 2:**
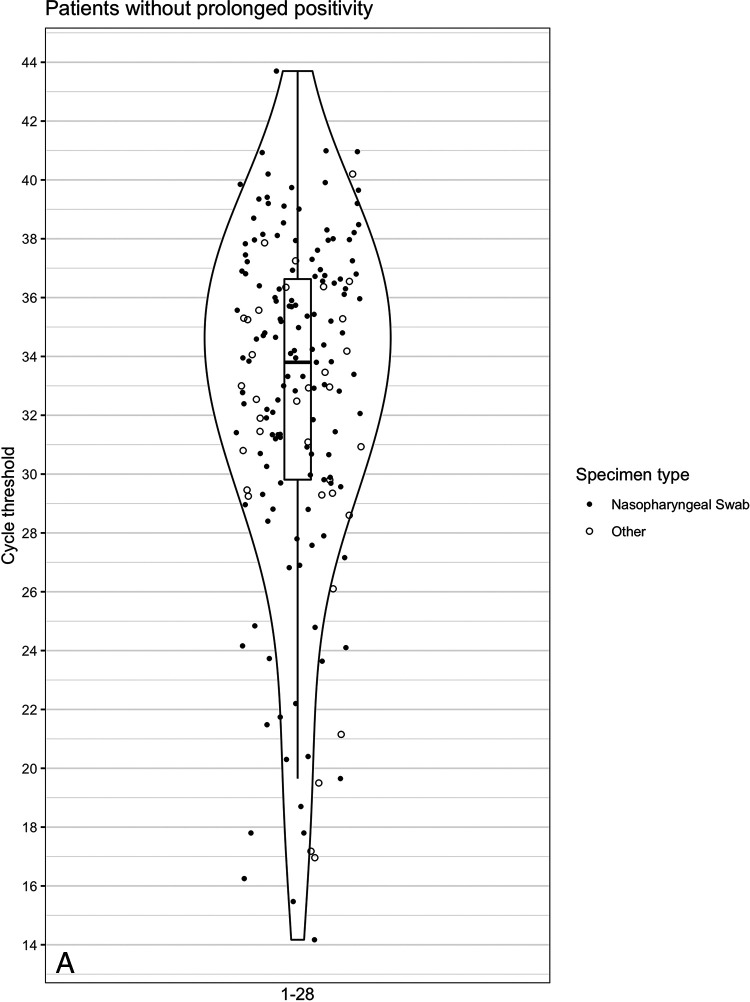

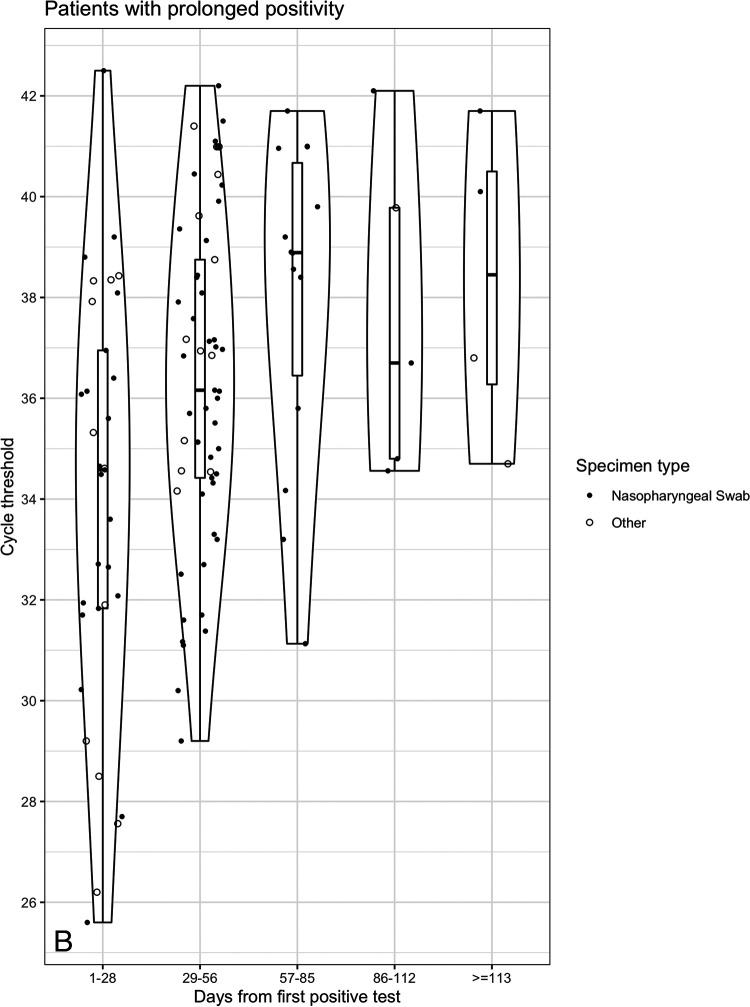
Distribution of *C_T_* values over time for patients with and without prolonged positivity. *x* axis indicates days between positive tests, and *y* axis indicates cycle threshold value of clinical tests. (A) Patients without prolonged positivity (<28 days). (B) Patients with prolonged positivity. Symbols indicate nasopharyngeal swab or other specimen type. Violin-and-box plot indicates distribution of *C_T_* values in time frames specified on the *x* axis.

While *C_T_* values generally increased over time, variable rates of increase in *C_T_* values were observed in individual patients (Fig. 2A and B; Fig. S2). Among patients without prolonged positivity, 123 (59%) had *C_T_* values available from both the first and last (terminal) positive test. Among these patients, terminal positive tests were a median of 9.8 cycles higher than first positive tests, signaling smaller amounts of RNA detected; however, 11 patients had larger amounts of RNA detected at their last positive test than at their first positive test, which occurred between 3 and 25 days prior. Overall, terminal positive tests ranged from 22.5 cycles lower to 31.8 cycles higher, with terminal positives testing near the limit of detection (>35) for 21 (17%) patients. For prolonged-positive patients’ terminal and initial tests, *C_T_* data were available for 36 patients (63%). Terminal positive tests were a median of 14 (range, 2 to 26) cycles greater than initial positive tests.

**FIG 3:**
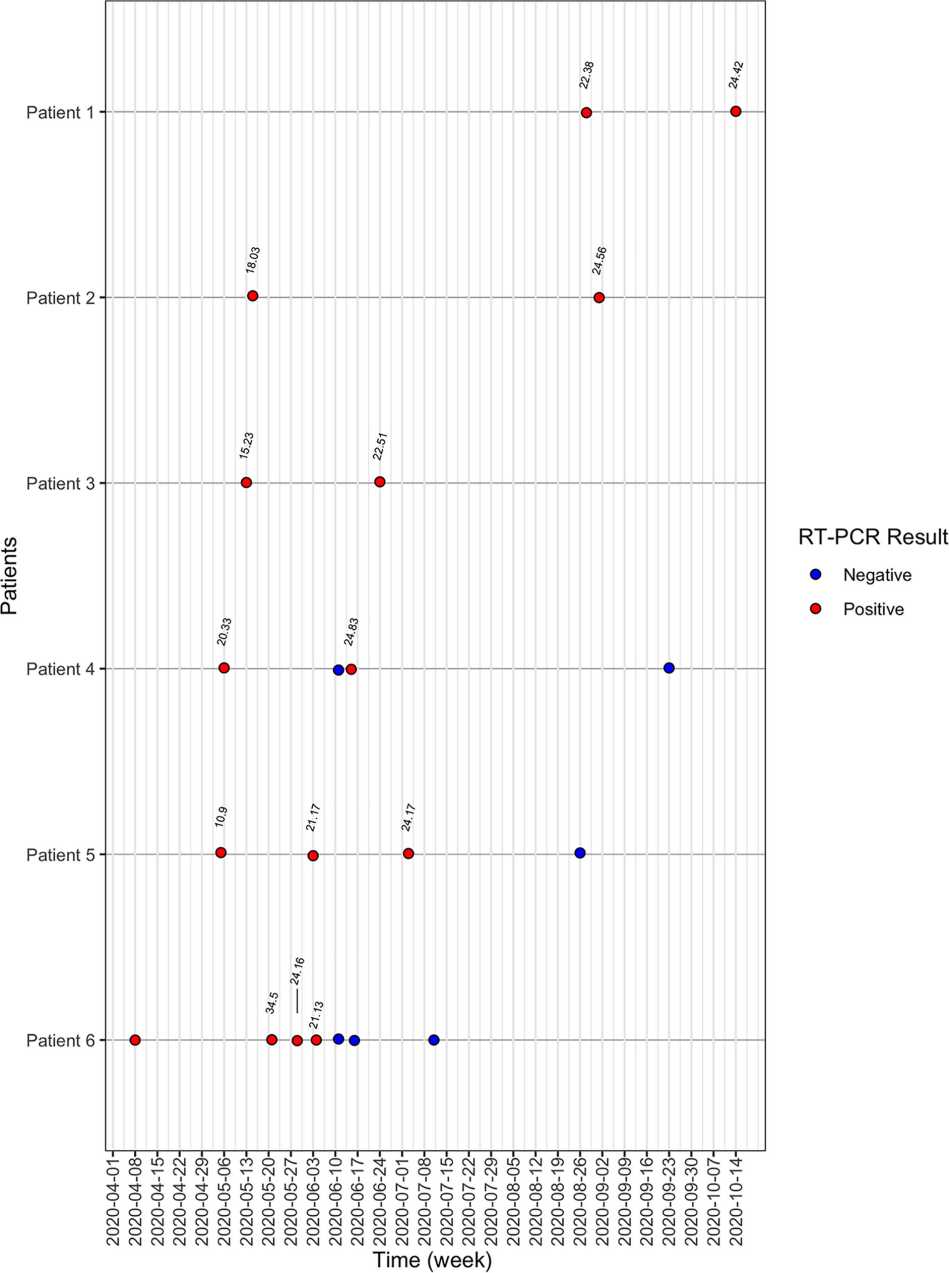
SARS-CoV-2 testing summary for patients with slow *C_T_* value increase, defined as *C_T_* values of <35 after 40 days post-initial positive test. *x* axis indicates time, and *y* axis indicates 1 patient per gray line. Red and blue dots indicate positive and negative SARS-CoV-2 tests, respectively. *C_T_* values are indicated.

In contrast to patients without prolonged positivity, no prolonged-positive patients had terminal test *C_T_* values that were lower than their initial positive test (i.e., an increase in the amount of RNA detected). Consistent with continued viral RNA decline over time, 33 (91%) prolonged-positive patients had terminal positive tests near the limit of detection (>35 *C_T_*); however, by day 40 after the initial positive test, 5 (13.8%) patients still had *C_T_* values of <35 and 1 patient tested positive with a *C_T_* value of <35 109 days after the initial positive test (Fig. 3).

### Demographic and treatment characteristics of prolonged-positive patients.

Patients with prolonged positivity are a population where the examination of *C_T_* values is an attractive method to help guide clinical decisions. The clinical characteristics of the 57 prolonged-positive patients included the following: obesity, 30%; hospitalization, 53%; discharge to long-term care, 35%; and hospice or deceased, 23%. In general, prolonged-positive patients experienced a severe course of illness, with 33% treated with immune-modulating drugs and 10% treated with remdesivir (Table 1).

**TABLE 1:** Clinical characteristics of patients with prolonged SARS-CoV-2 positivity

Characteristic	No. (%) of patients or mean value (±SD) (*n* = 57 patients)
Sex	
Female	31 (54)
Male	26 (46)
BMI (kg/m^2^)	31 (±9.8)
Missing	25 (43.9)
Age (yrs)	49 (±20)
Missing	1 (1.8)
First positive *C_T_* value	23 (±6.0)
Missing	13 (22.8)
Last positive *C_T_* value	37 (±3.2)
Missing	11 (19.3)
Time between first and last positive result (days)	51 (±27)
Admission	
Admitted	30 (53)
Never admitted	27 (47)
Discharge disposition	
Assisted living	2 (4)
Deceased	1 (2)
Home	17 (30)
Hospice	1 (2)
Never admitted	27 (47)
Rehab	1 (2)
Skilled nursing facility	6 (11)
Missing	2 (3.5)

## DISCUSSION

Here, we describe the natural history of SARS-CoV-2 RNA testing among all patients who tested positive multiple times at our institution during a 7-month period, revealing complex patterns of intermittent positivity and variable decline of viral RNA detected via *C_T_* values throughout different stages of infection. Although factors observed among prolonged-positive patients were similar to those described in patients with severe COVID-19 disease, 47% of prolonged-positive patients were not ill enough to require hospitalization, underscoring that a large subset of healthier patients may experience prolonged RNA positivity (1, 13). Intriguingly, 11 patients without prolonged positivity demonstrated greater amounts of RNA detected at their terminal versus initial SARS-CoV-2 positive test, suggesting variable shedding even early in their infection (2). Although our equivalency analysis showed limited variation in *C_T_* values across the platforms used in this study, and this was substantially lower than the variation in *C_T_* values at and between the study patients’ first and last positive tests (Table 1; Fig. S3, Table S1), some variation in RNA detected could be due to assay differences across platforms, although our results suggest this is effect is unlikely to change an observed negative or positive test result among the assays used in our study. Additional factors, such as timing of initial presentation and specimen quality, likely contribute to RNA detection or observed decline (2, 5, 14, 15). Although it was beyond the scope of this study to evaluate the likelihood of reinfection, which would require longitudinal viral sequencing, the observation of decreased RNA detection over time in the majority of patients was consistent with a single infection course, since reinfection is currently thought to be rare, especially prior to widespread VOC circulation (16).

The observation of intermittent positivity in 8% of patients with multiple SARS-CoV-2 positive tests during a time period prior to sustained VOC transmission suggests that sample quality may play a major role in *C_T_* results (14). Interestingly, prolonged-positive patients were over 3 times more likely to have intermittent positivity. These intermittent negatives may be explained by variable shedding dynamics, therapeutic interventions, or sample quality, among other variables, which will need to be investigated in future studies in order to better understand this phenomenon and how it pertains to infectivity and the clinical course of these patients.

As the pandemic progresses in time and VOCs capable of high-titer and vaccine breakthrough infections, such as was seen with Delta and Omicron, gain in prevalence, longitudinal testing information with variability in *C_T_* values will become available for a greater number of patients due to repeat testing for various reasons, including travel, exposures, and respiratory symptoms, many of which overlap other cocirculating respiratory viruses. With the added complexity of novel variant circulation, it will become even more crucial to keep the possibility of variable shedding in mind at any stage of infection and exercise caution when interpreting *C_T_* values as proxy measures for infectivity and severity in the absence of a patient’s full clinical picture, including physical examination, symptoms, and additional laboratory tests. Normalization of tests using international standards and deployment of diagnostic tests that can discriminate between prolonged shedding and reinfection and provide insight into infectiousness are urgently needed for the next stage of the SARS-CoV-2 pandemic.

## MATERIALS AND METHODS

### Study design and participants.

We performed a descriptive retrospective review of all patients who underwent testing for SARS-CoV-2 at the University of North Carolina Medical Center Clinical Microbiology Laboratory between 17 March 2020 and 15 October 2020. This study was approved by the Institutional Review Board at the University of North Carolina at Chapel Hill (IRB no. 20-2448).

### SARS-CoV-2 RNA testing.

During the study time frame, four platforms were used by our laboratory for the detection of SARS-CoV-2. Three of these assays, the Abbott Alinity m SARS-CoV-2 assay, Abbott RealTime m2000 SARS-CoV-2 assay (Abbott Molecular, Inc., Green Oaks, IL), and Cepheid Xpert Xpress SARS-CoV-2 (Cepheid, Sunnyvale, CA) had accessible cycle threshold data that were exported from the instruments. *C_T_* values were not available from an emergency use authorization (EUA) laboratory-developed test that was used on a minority of specimens. To adjust for cycle threshold reporting differences between instruments, 10 cycles were added to *C_T_* values from the Abbott m2000 assay to account for the 10 hidden cycles in the analysis software (Abbott Molecular, Inc., personal communication).

### Evaluation of *C_T_* normalization or *C_T_* value equivalency across testing platforms.

The validity of comparing *C_T_* results across platforms was assessed across a range of positive *C_T_* values using pooled remnant specimens across the range of high (33 to 35), middle (25 to 30), and low (15 to 20) *C_T_* values representing low, moderate, and large amounts of SARS-CoV-2, respectively. Pools were tested in triplicate across each of the three instruments on three different days. The reproducibility and variability of *C_T_* results were evaluated across pools and platforms using linear regression and comparison of mean values and standard deviations of *C_T_* values across pools, platforms, and test days.

### Clinical chart review and data abstraction.

Basic demographic information and SARS-CoV-2 test data (positive and negative tests, specimen type, testing platform, and *C_T_* values) were abstracted from electronic medical records (EMR) and the laboratory information system for all patients tested for SARS-CoV-2 during the study period. We queried these reports to identify patients with multiple positive tests and examined the timing of positive tests to identify patients who had evidence of long-term positivity of SARS-CoV-2. Patients who demonstrated positivity later than 28 days (3rd quartile of all patients with multiple positive tests) were considered “long-term”-positive patients and were investigated further by chart review. Manual chart review of the EMR was performed to abstract patient age, gender, body mass index (BMI), hospital admission dates, and discharge disposition. Medication exposure for long-term-positive patients was collected via EMR reports.

SARS-CoV-2 testing data were evaluated in relation to positive and negative testing dates to describe the characteristics of the patient population with prolonged positivity and to summarize patterns of clinical exposures surrounding dates when patients either tested negative or positive for SARS-CoV-2.

### Statistical analysis.

All descriptive statistics and data analysis were performed using R version 3.6.1 (17).
