# Supplementary material for: Longitudinal SARS-CoV-2 Testing among the Unvaccinated Is Punctuated by Intermittent Positivity and Variable Rates of Increasing Cycle Threshold Values
Source: Microbiol Spectr. 2022 Mar 22;10(2):e02715-21. doi: 10.1128/spectrum.02715-21 (PMC9045176; doi:10.1128/spectrum.02715-21)
Supplement: SUPPLEMENTAL FILE 1 — Supplemental material. Download SPECTRUM02715-21_Supp_1_seq8.pdf, PDF file, 0.6 MB [file spectrum02715-21_supp_1_seq8.pdf]

Supplemental Information

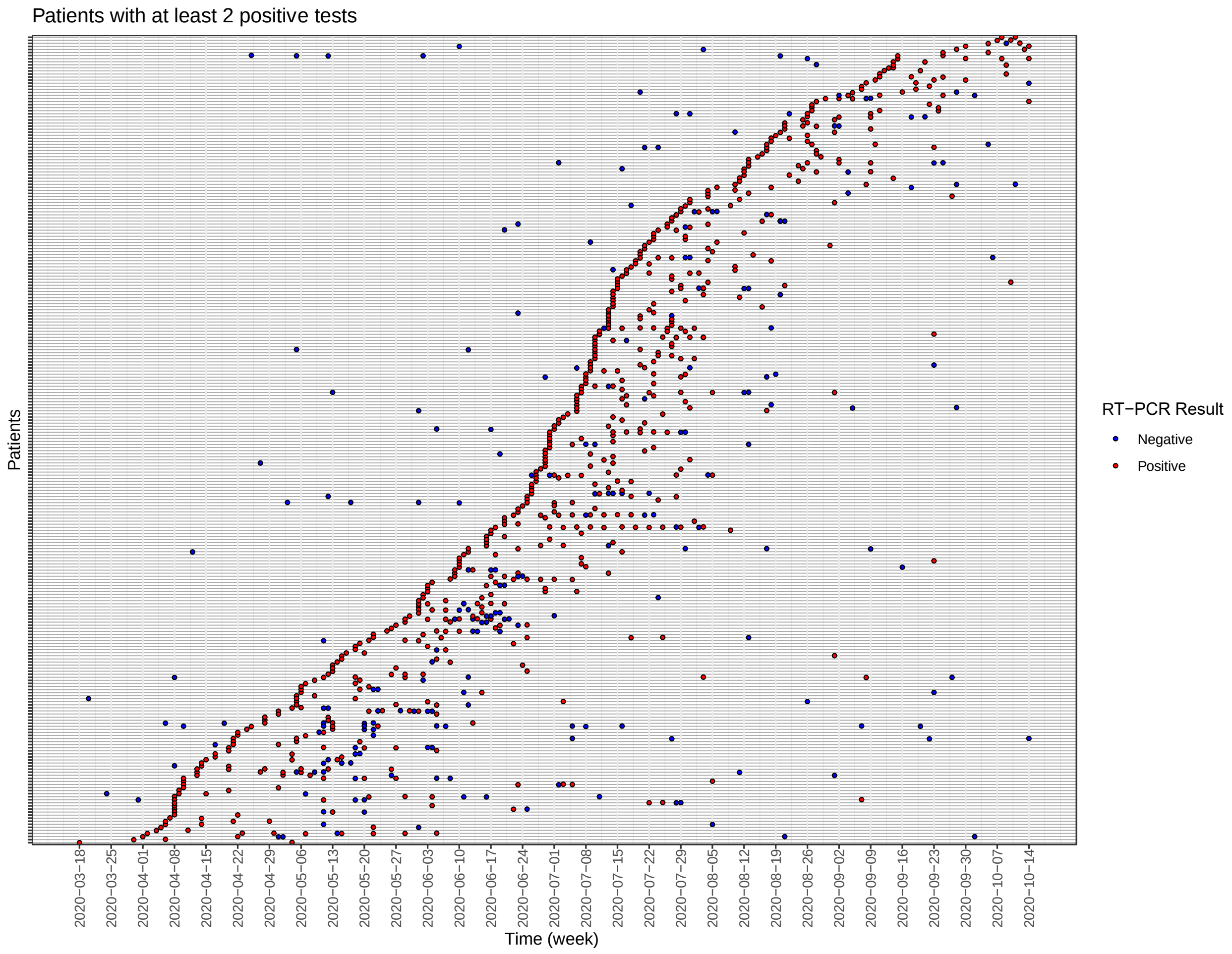

Figure S1. Timeline of SARS-CoV-2 testing throughout study for patients with at least 2 positive SARS-CoV-2 tests. X-axis indicates time, y axis indicates 1 patient per grey line. Red and blue dots indicate positive and negative SARS-CoV-2 tests respectively.

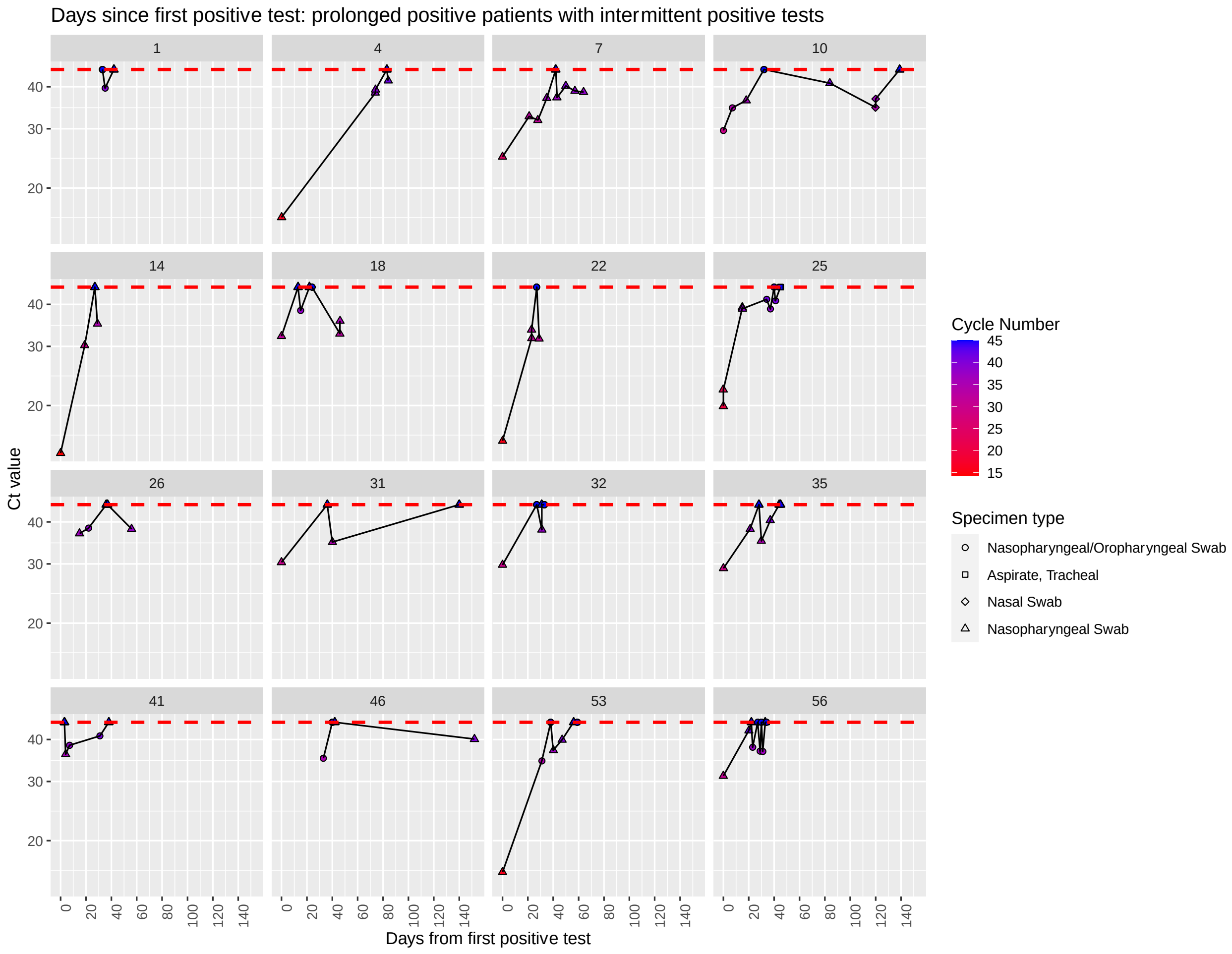

Figure S2. Timeline of Ct values for prolonged positive patients with intermittent positive and negative tests. Y axis indicates Ct value, X axis indicates days from first positive test. Patients are represented by number in grey box at top of each plot. Red line indicates negative test. Tests are only plotted if negative or positive and a Ct value was available.

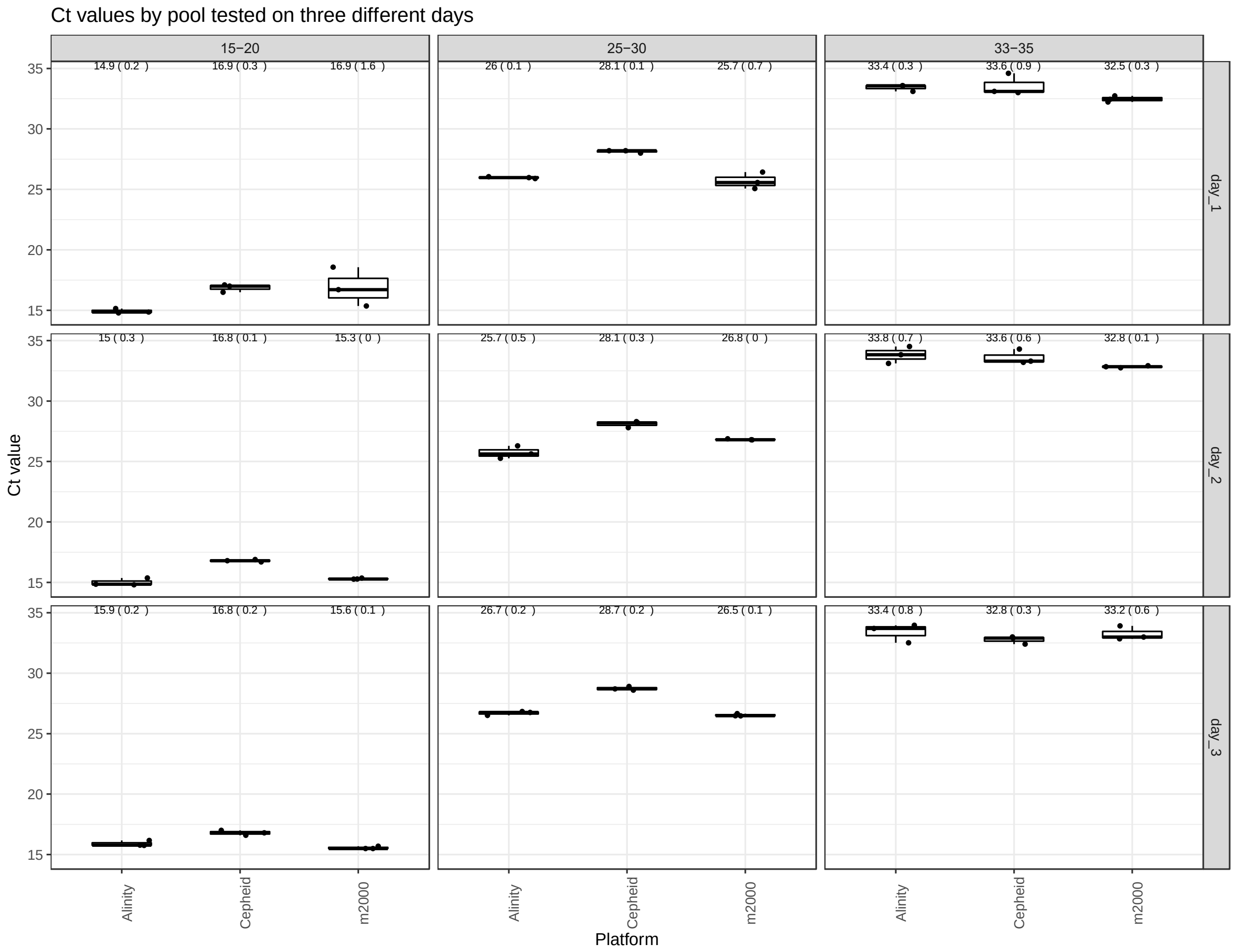

Figure S3. Consistency of Ct value results across platforms over the full range of Ct values for positive tests. Assessment of comparability across a range of Ct values and testing platforms was made using pooled remnant positive samples with original Ct values in the range specified across top grey boxes. Ct values (left y-axis) of pools tested in triplicate on each platform (x-axis) on three days (right y-axis grey boxes) showed highly reproducible Ct value results with limited variation across starting material ranges and platforms. The mean and standard deviation (mean (SD)) across triplicate pool results on the day the pooled material was tested are listed at the top the box above each corresponding platform.

Table S1. Summary of equivalency analysis combining triplicate RT-PCRs over three days.

Platform	Pool Range (Ct)	Mean (Ct)	SD
m2000	15-20	15.92	1.09
Alinity	15-20	15.28	0.51
Cepheid	15-20	16.82	0.20
m2000	25-30	26.35	0.62
Alinity	25-30	26.13	0.52
Cepheid	25-30	28.32	0.35
m2000	33-35	32.85	0.46
Alinity	33-35	33.54	0.58
Cepheid	33-35	33.31	0.70
